# Taeniasis impacts human gut microbiome composition and function

**DOI:** 10.1093/ismejo/wrae213

**Published:** 2024-10-23

**Authors:** Wenjie Mu, Pingping Ma, Yugui Wang, Yaqi Li, Yingying Ding, Yang Zou, Lixia Pu, Qi Yan, Haoyue Kong, Xiaola Guo, Aijiang Guo, Hailong Li, Shuai Wang

**Affiliations:** State Key Laboratory of Animal Disease Control and Prevention, College of Veterinary Medicine, Lanzhou University, Lanzhou Veterinary Research Institute, Chinese Academy of Agricultural Sciences, Lanzhou, Gansu 730000, China; Key Laboratory of Veterinary Parasitology of Gansu Province, Lanzhou Veterinary Research Institute, Lanzhou, Gansu 730046, China; State Key Laboratory of Animal Disease Control and Prevention, College of Veterinary Medicine, Lanzhou University, Lanzhou Veterinary Research Institute, Chinese Academy of Agricultural Sciences, Lanzhou, Gansu 730000, China; State Key Laboratory of Animal Disease Control and Prevention, College of Veterinary Medicine, Lanzhou University, Lanzhou Veterinary Research Institute, Chinese Academy of Agricultural Sciences, Lanzhou, Gansu 730000, China; State Key Laboratory of Animal Disease Control and Prevention, College of Veterinary Medicine, Lanzhou University, Lanzhou Veterinary Research Institute, Chinese Academy of Agricultural Sciences, Lanzhou, Gansu 730000, China; State Key Laboratory of Animal Disease Control and Prevention, College of Veterinary Medicine, Lanzhou University, Lanzhou Veterinary Research Institute, Chinese Academy of Agricultural Sciences, Lanzhou, Gansu 730000, China; State Key Laboratory of Animal Disease Control and Prevention, College of Veterinary Medicine, Lanzhou University, Lanzhou Veterinary Research Institute, Chinese Academy of Agricultural Sciences, Lanzhou, Gansu 730000, China; State Key Laboratory of Animal Disease Control and Prevention, College of Veterinary Medicine, Lanzhou University, Lanzhou Veterinary Research Institute, Chinese Academy of Agricultural Sciences, Lanzhou, Gansu 730000, China; State Key Laboratory of Animal Disease Control and Prevention, College of Veterinary Medicine, Lanzhou University, Lanzhou Veterinary Research Institute, Chinese Academy of Agricultural Sciences, Lanzhou, Gansu 730000, China; State Key Laboratory of Animal Disease Control and Prevention, College of Veterinary Medicine, Lanzhou University, Lanzhou Veterinary Research Institute, Chinese Academy of Agricultural Sciences, Lanzhou, Gansu 730000, China; State Key Laboratory of Animal Disease Control and Prevention, College of Veterinary Medicine, Lanzhou University, Lanzhou Veterinary Research Institute, Chinese Academy of Agricultural Sciences, Lanzhou, Gansu 730000, China; Key Laboratory of Veterinary Parasitology of Gansu Province, Lanzhou Veterinary Research Institute, Lanzhou, Gansu 730046, China; State Key Laboratory of Animal Disease Control and Prevention, College of Veterinary Medicine, Lanzhou University, Lanzhou Veterinary Research Institute, Chinese Academy of Agricultural Sciences, Lanzhou, Gansu 730000, China; Key Laboratory of Veterinary Parasitology of Gansu Province, Lanzhou Veterinary Research Institute, Lanzhou, Gansu 730046, China; Department of Parasitology, College of Basic Medicine, Dali University, Dali, Yunnan 671000, China; State Key Laboratory of Animal Disease Control and Prevention, College of Veterinary Medicine, Lanzhou University, Lanzhou Veterinary Research Institute, Chinese Academy of Agricultural Sciences, Lanzhou, Gansu 730000, China; Key Laboratory of Veterinary Parasitology of Gansu Province, Lanzhou Veterinary Research Institute, Lanzhou, Gansu 730046, China

**Keywords:** tapeworm, gut microbiome, *Bifidobacterium*, stachyose metabolism

## Abstract

Human taeniasis, caused by *Taenia* tapeworms, is a global parasitic disease with significant implications for public health and food safety. These tapeworms can grow to considerable sizes and potentially impact the microecology of the host gut. Despite their importance, the effects of *Taenia* infection on host gut microbiota haven’t been thoroughly investigated. In this study, we conducted a cross-sectional analysis of the gut microbiome in patients infected with *Taenia asiatica* (*n* = 87) compared to healthy controls (*n* = 79) in the Dali cohort, China. We also performed a longitudinal assessment of microbial changes following deworming in a subset of patients (*n* = 24). Our findings reveal a significant shift in gut microbial composition, characterized by increased alpha-diversity and an enrichment of *Prevotella*-driven enterotypes in infected patients compared to healthy controls. The stability of these microbial features post-deworming varied widely among individuals and was lower in those with lower initial alpha diversity and *Prevotella*-enterotype before deworming. We observed a significant depletion of *Bifidobacterium* species in infected individuals, regardless of enterotypes, and these prebiotics did not recover post-deworming. Metabolic network analysis and *in vitro* experiments suggest that the reduction of *Bifidobacterium* was linked to metabolic competition for ecological niches or nutrients, particularly stachyose, from other microbes rather than the parasitism itself. Furthermore, our machine learning analysis demonstrated that taxa associated with *Bifidobacterium* in stachyose metabolism could robustly predict infection but could not predict deworming. This study highlights the substantial impact of taeniasis on the human gut microbiome and overall gut health.

## Introduction

Human taeniasis is an intestinal infection caused by tapeworms from the genus *Taenia*. Infection occurs through the consumption of undercooked beef (*T. saginata*, beef tapeworm) or pork (*T. solium*, pork tapeworm; *T. asiatica,* Asian tapeworm, a sister species of *T. saginata*) [1]. Humans are the only definitive hosts for these tapeworms, whereas cattle and pigs serve as intermediate hosts [2]. It is estimated that ~100 million people are infected with *Taenia* annually [3], which causes a significant problem for public health and food safety.

The most visible sign of taeniasis is the passage of proglottids (tapeworm segments) in feces. Although many infections are asymptomatic, the large size of *Taenia* tapeworms—typically ranging from 3 to 10 m—can lead to digestive issues. Symptoms may include weight loss, abdominal pain, loss of appetite, diarrhea, and, in severe cases, ileum bleeding [4, 5]. These observations suggest that tapeworm infections can significantly impact the human digestive system. Despite their high prevalence, the full extent of their effects on human health is often underestimated.

Evidence suggests that many intestinal parasites, including protozoa and helminths, can profoundly impact the composition of the gut microbiota [6–8]. Both parasites and the gut microbiota compete for similar ecological niches within the host. Additionally, the presence of parasites may trigger the host’s immune response, disrupting the homeostatic balance between the bacterial microbiota and its host [9, 10]. Although helminths were once regarded solely as pathogenic, recent studies have shown that they may also have commensal or even beneficial effects on the host [9, 11]. Epidemiological research reveals that populations with high rates of helminth infections often exhibit lower incidences of autoimmune diseases and allergic conditions [12]. Recent findings suggest that the gut microbiota may play a role in mediating the beneficial effects of helminths by modulating the host’s immune responses and alleviating inflammation, such as by promoting regulatory T cells and alternatively activated macrophages [12–14]. Given the critical role of the gut microbiota in human health and disease, understanding whether and how a helminth infection alters gut microbiota is essential for making informed medical decisions.

Despite the significant impact of *Taenia* infection on the host health, its effects on the host gut microbiota and how it recovers post-deworming remain largely unexplored. In this study, we examined a cohort of *Taenia*-infected patients and healthy controls (HCs) in Dali, China, to investigate the impact of *Taenia* infection on the human gut microbiota and to assess how the parasite affects the commensal microbes in the host intestine. Our findings suggest that *Taenia* infection significantly alters the human gut microbiota, particularly by reducing prebiotic species potentially due to metabolic competition between microbes. Additionally, the effects of deworming on gut microbiota appear to be dependent on the specific gut content of the individuals.

## Materials and methods

### Ethics

This study has been approved by the Medical Ethics Committee of Dali University (MECDU-202010-1). All participants signed an informed consent agreement before donating their fecal samples.

### Study cohorts and fecal sample collection

The fecal samples of patients were collected from 2020 to 2023 in Dali (Yunnan province, China). For each patient, we recorded their age, gender, height, weight, diet, and medication information to ensure they were undergoing deworming for the first time and had not received any medication prior to deworming. Fecal samples were collected with the patient’s permission before administering anthelmintics. As Dali is a pandemic region of asymptomatic neurocysticercosis, the administration of praziquantel has a risk of induction of epileptic seizures or convulsions [15]. After evaluation by clinicians, the specific anthelmintic regimen and procedures based on a traditional remedy with a combination of pumpkin and betel nut were performed using methods as previously reported [16] (Supplementary information). For the patients who agreed to participate in the longitudinal study, the fecal samples were also collected through a follow-up study at a time point from 3 to 12 months post-deworming. HCs were recruited from the same region and matched with the patients for gender and age. Each fecal sample was divided into multiple parts and stored at −80°C until use. Stool DNA was extracted using the Stool DNA Extraction Kit (D4015-00 E.Z.N.A.® Stool DNA Kit, OMEGA, USA).

Participants with severe chronic diseases (i.e. diabetes, HIV, and cancers), chronic, or viral liver diseases (i.e. cirrhosis, MASH, HBV, and HCV), gastrointestinal (GI) diseases (i.e. IBD and IBS), or those who had taken antibiotics or antifungal medications within the last 3 months were excluded from both patient and control groups. We used the *COX I* gene to re-identify fecal samples from patients infected with *T. asiatica* [17]. Traditional microscopic observation methods were applied to stools from HCs to filter out samples containing eggs from other worms [18, 19]. In addition, a method based on PCR amplification was used to further exclude infections of other worms. Six helminth species (*Ascaris lumbricoides*, *Strongyloides stercoralis*, *Trichuris trichiura*, *Ancylostoma* spp., *Necator americanus*, and *Fasciola* spp.) were screened by qPCR or PCR (for *Fasciola* spp.) according to the methods described [18, 19]. Samples from both groups that contained these listed pathogens were excluded for further analyses.

### Metagenomic sequencing and analyses

DNA libraries were then prepared using the Rapid Plus DNA Lib Prep kit (RK20208, Illumina), following the manufacturer’s recommendations. Sequencing was performed on the NovaSeq 6000 platform (Illumina). The raw shotgun sequencing reads were processed using the bioBakery meta’omics workflow to generate taxonomic and functional profiles. Specifically, the raw paired-end reads (PE-150) were processed with the KneadData (v0.10.0) quality control pipeline (https://huttenhower.sph.harvard.edu/kneaddata/), using the parameters: -t 30 --trimmomatic-options “ILLUMINACLIP: TruSeq3-PE-2.fa:2:30:10:8 LEADING:3 TRAILING:3 MINLEN:50 SLIDINGWINDOW:5:20”. This step removed barcodes, low-quality reads, and host-origin reads (human, GRCh38).

Taxonomic annotation was performed using MetaPhlAn4 (v4.0.3) [20] with the standard reference database (mpa_vJan21_CHOCOPhlAnSGB_202103). Functional annotation and pathway composition were determined using HUMAnN3 (v3.6) [21] based on the UniRef90 and MetaCyC databases with default parameters. The taxa with differential abundances between groups were identified using the Analysis of Compositions of Microbiomes (ANCOM) method, implemented in R package ANCOMBC (v2.20) [22]. R package MaAsLin2 (v1.73) [23] was employed for differential analysis of microbiota pathways, respectively. The β-diversity was estimated by Bray–Curtis distance and significance levels were determined using permutational multivariate analysis of variance (PERMANOVA) with the “Adonis2” function in R package vegan (v2.6-4) [24]. The centered log-ratio (CLR) transformation of compositional data was performed by R package compositions (v2.0-4) (http://www.stat.boogaart.de/compositions/). The Procrustes test was performed by R package vegan (v2.6-4) [24]. The Orthogonal partial least squares discriminant analysis (OPLS-DA) of metabolites was performed by R package ropls (v1.37.0) [25]. The growth rates of microbiota were assessed by the growth rate index (GRiD, v1.3) using metagenomic data. Samples were mapped to a standard stool-specific GRiD database and calculated growth rate of each bacteria using GRiD software [26].

### Enterotype analyses

For the enterotype analysis, the gut bacteria components were clustered based on all the samples at the genus level according to the method described previously [27]. Briefly, the bacterial taxa that are present in <20% of total samples were excluded to decrease noise. Then, the samples were clustered using partitioning around medoid (PAM) based on Jensen–Shannon distance (JSD) between samples. The Calinski–Harabasz index was used to estimate the optimal number for PAM clusters. Between-class analysis (BCA) was performed to validate the clustering results and detect the dominant bacteria for each cluster.

### Random forest (RF) analysis

To develop a microbial-based prediction model capable of distinguishing infected and healthy individuals, we implemented random forest (RF) methods using R package randomForest (v4.7-1.1) to generate a microbiota RF classifier as reported in the previous study [28]. Briefly, the original dataset was randomly partitioned into a training (discovery) dataset and a validation dataset with a proportion of 7:3 for all the samples. The optimal number of markers for classifying disease and health within the training dataset was determined by a 10-fold cross-validation method with five independent replicates using parameters “importance = TRUE”. The point with the minimum cross-validation error was viewed as the cutoff point and the features before this point were selected as an optimal set of markers based on the ranked value of MeanDecreaseGini. The statistical significance (*P* value) for each marker was calculated by R package rfPermute (v2.5.2) (https://github.com/EricArcher/rfPermute). Only markers with *P* values <.05 in the permutation test were kept. The final “bagged” RF classifier based on the identified optimal set of microbial features was subsequently utilized to assess the predictive efficiency in both the training and validation datasets. The performance of each classifier was evaluated using the receiver operating characteristic (ROC) curve, implemented in R package pROC (v1.18.4) [29].

### Metabolomic analysis

For the metabolomic analysis, the participants in the disease group were randomly selected from our original cohort and the relevant HCs were selected to match the potential confounding factors, including sex, age, and BMI. The preparations of the samples for UHPLC-HRMS-based metabolomic analysis were conducted using the method previously reported [30]. The details are shown in Supplementary Information. The raw data were converted to MzXML files using ProteoWizard MSConvert [31] before being imported into XCMS software [32]. For the extracted ion features, only the variables with more than 50% non-zero measurements in at least one group were retained for analysis. Metabolite identification was carried out using an in-house database constructed from available authentic standards. Following normalization to the total peak intensity, the processed data underwent multivariate data analysis. The variable importance in the projection (VIP) value in the OPLS-DA model was calculated to assess its contribution to classification.

### Network analysis

The microbiota-metabolite network was constructed and annotated by R package MetaNet (v0.13) (https://github.com/Asa12138/MetaNet) using the Spearman correlation coefficient. Only the network between differentially abundant bacteria and differential metabolites was analyzed by c_net_build function with parameter “r_thres = 0, p_thres = 0.05, delete_single = T”. Modules were clustered with module_detect function with parameter “n_node_in_module = 10, delete = T”.

### Bacteria culturing experiments

The *Dorea longicatena* strain (DSM13814, DSMZ, German), *B. longum* strain (ATCC15707, Mingzhoubio, China), and *Escherichia coli* O157:H7 strain (ATCC700728, Biobw, China) were purchased from bioresource centers and cultured in modified PYG medium (DSMZ), GAM medium (Cat. HB8518, HopeBio, China), and LB medium at 37°C in anaerobic condition, respectively. Herein, a widely prevalent pathogenic *E. coli* strain was used as the control for the beneficial bacterium *B. longum*. The live tapeworms were isolated from dewormed patients and cultured in PBS and the culture supernatants were collected after centrifugation at 4000 × *g* for 10 min at 4°C. The excretory-secretory (ES) proteins were concentrated with a Millipore ultrafiltration centrifuge tube (Cat. 23227, Thermo Fisher, USA) at 4000 × *g* at 4°C. The protein concentration of the concentrated ES proteins was evaluated using the BCA Protein Assay Kit (Cat. P0012S, Beyotime, China) according to the manufacturer’s protocol. Each bacterium was cultured, harvested at the exponential phase (with an OD600 value of 0.06), then inoculated in fresh culture medium (1 × 10^5^ CFU/cell), and cultured on 96-well cell culture plates in the presence of ES proteins or stachyose (Cat. B21193, Yuanye Bio, China) for 24 h to assess their effects on growth. The OD600 value was measured every 4 h by a spectrophotometer (SpectraMax ABS Plus, Molecular Devices) for the culture medium at each concentration (ES proteins or stachyose) (*n* = 4 replicates per sample).

### Statistical analysis

Statistical analyses were conducted using R (v4.4.0). The Wilcoxon rank-sum test (two-sided; confidence level of 0.95) was employed to compare the difference of specific taxa, pathways, alpha diversities, and metabolite concentrations between groups. The Benjamini–Hochberg procedure (FDR) was used to correct *P* values for multiple hypothesis testing. Spearman correlation coefficient was used to calculate the correlation between two variables.

## Results

### Cohort description

**Figure 1:**
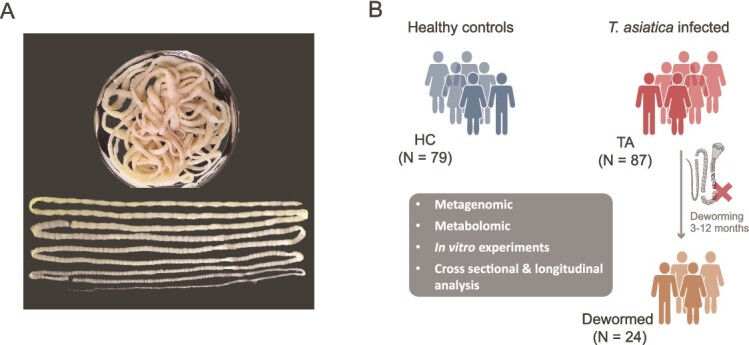
The overview of the study’s design. (A) Morphology of the human tapeworm *Taenia asiatica*. (B) Study overview depicting the study cohorts (cross-sectional and longitudinal) and methodological approaches, including metagenomic, metabolomic, and *in vitro* experiments.

To investigate the impact of *Taenia* infection on the human gut microbiome, we recruited patients with *T. asiatica* infection (Fig. 1A) from Dali, China, a region with a high prevalence of tapeworm infections. Diagnosis of taeniasis was confirmed through the detection of proglottids in feces and identification of the parasites via *COXI* gene analysis. We also recruited age- and sex-matched HCs from the same region. All participants, both infected and healthy, had a diet consisting of balanced plant and animal foods and no other significant underlying diseases. To control for the influence of other intestinal helminths, we screened for common parasites-*Ascaris lumbricoides* (roundworm), *Strongyloides stercoralis* (roundworm), *Trichuris trichiura* (whipworm), *Ancylostoma* spp. (hookworm), *Necator americanus* (hookworm), and *Fasciola* spp. (flatworm)—using microscopic examination and qPCR or PCR [18, 19]. Samples from both the infected and healthy groups with any identified helminth infections were excluded from further analysis. After this screening, the *Taenia*-infected group (TA) comprised 87 subjects and the HC group included 79 subjects (Table S1). Additionally, 24 patients agreed to participate in a follow-up study conducted 3–12 months post-deworming (Fig. 1B and Fig. S1). All samples underwent metagenomic shotgun sequencing, producing at least 4.65 Gb of high-quality data per sample. This comprehensive cohort allowed us to both cross-sectionally and longitudinally characterize the gut microbiome alterations associated with *T. asiatica* infection.

### Gut microbial community and enterotypes are markedly altered in taeniasis

**Figure 2:**
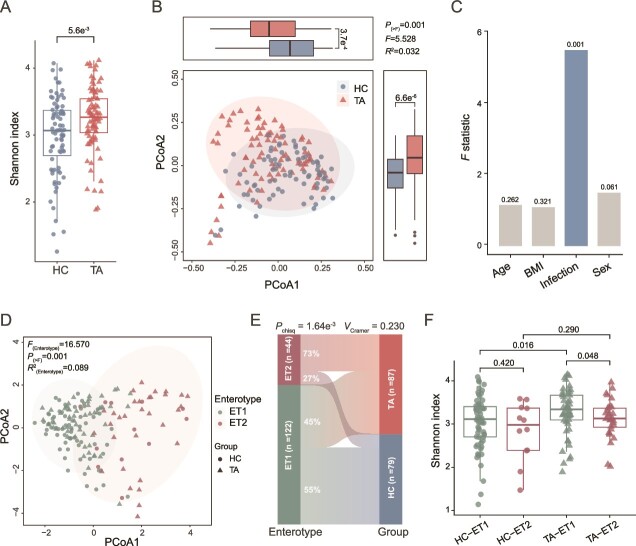
The alterations in the gut microbiome in infection of *T. asiatica*. (A) The comparison of the Shannon Index between the health control (HC) (*n* = 79) and *T. asiatica-*infected (TA) (*n* = 87) groups. (B) The species level’s Principal Coordinates Analysis (PCoA) based on Bray–Curtis distances. (C) Effect sizes from PERMANOVA analysis, represented by *F* statistics, for each variable (age, BMI, infection status, and sex), respectively. The *P* value for each variable on gut microbiota variation was shown above the bar. (D) PCoA of the enterotypes for the gut microbiome. Enterotypes were identified using JSD and PAM clustering at the genus level. (E) The links between enterotype (ET1 and ET2) and infection status (HC and TA). (F) Comparison of the Shannon index within enterotypes (ET1 and ET2) across HC and TA groups. The *P* values for panels A and F were determined by the Wilcoxon rank-sum test (two-sided). The *R*^2^ statistic and *P* values for panels B and D were calculated by permutational multivariate analysis of variance (PERMANOVA). The correlation in panel E was measured by carmer’s *V*, and the *P* value was calculated by the chi-square test. The box plot represents the 25th percentile, median, and 75th percentile and whiskers stretch to 1.5 times the interquartile range from the corresponding hinge.

To investigate alterations in the gut microbiota associated with taeniasis, we analyzed alpha and beta diversities at the species level. We found that the alpha diversity (Shannon Index) was significantly higher in the TA group compared to the HC group (Fig. 2A). Beta-diversity analysis, using Principal Coordinates Analysis (PCoA) based on Bray–Curtis distances, showed a significant shift in microbiota compositions (Fig. 2B). Adonis analysis further indicated that *T. asiatica* infection was the primary factor driving variations in host gut microbiota, surpassing other covariates, such as sex, BMI, and age (Fig. 2C). At the phylum level, the gut microbiota compositions differed between infected and healthy individuals, with notable alterations in Actinobacteria (Fig. S2). These findings are consistent with previous research on other helminth infections [33] and suggest that *T. asiatica* infection has a substantial impact on the gut microbial community in humans.

We also examined variations in gut microbial patterns in terms of enterotypes. Using JSD-PAM methods, we classified the microbiota into two enterotype clusters, characterized by the dominant genus: either *Faecalibacterium* (ET1) or *Prevotella* (ET2) (Fig. 2D and Fig. S3). Enterotypes ET1 and ET2 represented 73% and 27% of all the samples, respectively. PCoA based on Jensen–Shannon divergence demonstrated a significant distinction between samples from ET1 and ET2 (Fig. 2D). ET2 was enriched in individuals with *T. asiatica* infection, whereas ET1 was more prevalent among healthy individuals (Fig. 2E). Although gut microbial diversity was comparable between the two enterotypes in healthy individuals, ET1 in infected individuals exhibited measurably higher diversity compared to ET2 (Fig. 2F). These results support the notion that the gut microbiota structure is remodeled during *T. asiatica* infection.

### Microbiome stability after deworming depends on alpha diversity and enterotype in infection

**Figure 3:**
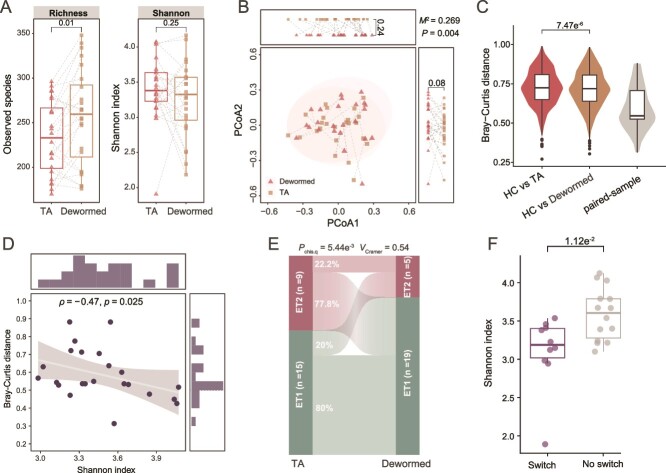
The landscape of the gut microbiome in patients post-deworming. (A) The paired comparison of alpha diversity indices (Richness and Shannon) between baseline and followed-up samples from each individual in longitudinal analysis (*n* = 24). (B) The species level’s PCoA based on Bray–Curtis distances. (C) The sample Bray–Curtis distances of inter-groups (HC vs. TA; HC vs. Dewormed) and paired samples (baseline vs. post-deworming, pairwise). (D) The correlation between the baseline’s Shannon Index and paired Bray–Curtis distances between baseline and post-deworming samples from the same individual. (E) Distribution of enterotypes in samples of baseline and post-deworming. (F) Comparisons of alpha diversity (Shannon Index) between samples with (*n* = 10) and without (*n* = 14) enterotype shifts following deworming. The *P* values for panels A and C were determined by the paired Wilcoxon rank-sum test. The M2 statistic and *P* value for panel B were calculated by Procrustes analysis and permutations test, respectively. The correlation for panel D was calculated by Spearman rank correlation coefficient. The correlation for panel E was measured by carmer’s *V* and the *P* value was calculated by the chi-square test. The *P* values for panel F were determined by an unpaired Wilcoxon rank-sum test. The box plot represents the 25th percentile, median, and 75th percentile and whiskers stretch to 1.5 times the interquartile range from the corresponding hinge.

We investigated changes in the host gut microbiota following the clearance of infection using longitudinal data. Post deworming, we observed a significant increase in species richness, although the Shannon Index showed no differences (Fig. 3A). The overall microbial community did not exhibit significant shifts between the pre- (baseline) and post-deworming groups, as indicated by PCoA results based on Bray–Curtis distances (Fig. 3B). This suggests that deworming leads to subtle changes in the gut microbiota of infected patients. However, when comparing with Bray-Curtis distances to healthy individuals, a significantly measurable decrease was observed in these patients (Fig. 3C), suggesting that their gut microbial structure was similar to that of healthy individuals. However, this alteration was not associated with the sampling interval (Fig. S4). Variation in microbial stability was observed among individuals, with many of them (79%) showing high Bray–Curtis distances (>0.5) between pre- and post-deworming samples, whereas others (21%) showing low distances (<0.5) (Fig. 3D). This variation was not correlated with the sampling interval (Fig. S5), indicating that the stability of the gut microbiome post-deworming may be individually dependent.

We further analyzed the factors linked with the individual stability of gut microbiota in deworming. Patients with a higher baseline Shannon Index exhibited smaller within-individual Bray–Curtis distances post-deworming (Fig. 3D). This suggests that higher alpha diversity in infection is associated with a more stable microbiome after infection clearance. Further analysis revealed that baseline abundances of 11 microbial species, including *Roseburia inulinivorans*, *Dorea longicatena*, *Lachnospira eligens*, and *Bacteroides nordii*, were significantly correlated with Bray–Curtis distances between samples of baseline and post-deworming within individuals (Table S2). These findings indicate that gut microbial stability after deworming varies among individuals and is associated with baseline alpha diversity.

Given that individuals with higher diversity have fewer microbiota changes post-deworming, we hypothesized that patients with the ET1 enterotype before deworming might have a more stable microbiome compared to those with the ET2 enterotype. Our analysis confirmed this hypothesis: 41.7% of dewormed samples switched enterotypes; this switch was accompanied by a significant change of the dominant genus *Faecalibacterium* and *Prevotella* (Fig. S6). Specifically, 20.0% of ET1 samples transitioned to ET2, whereas 77.8% of ET2 samples shifted to ET1 (Fig. 3E). This supports the notion that the infection-associated *Prevotella* (ET2) enterotype is more likely to change after deworming. This enterotype instability was not affected by the sampling interval (Fig. S7). Additionally, we found that the baseline Shannon or Simpson Index of samples with an enterotype switch after deworming was significantly lower than samples without a switch (Fig. 3F). Collectively, these results suggest that microbial diversity and enterotype during infection are linked to the stability of the host gut microbiota after deworming.

### Reduction of *Bifidobacterium* features infected and dewormed patients irrespective of enterotypes

**Figure 4:**
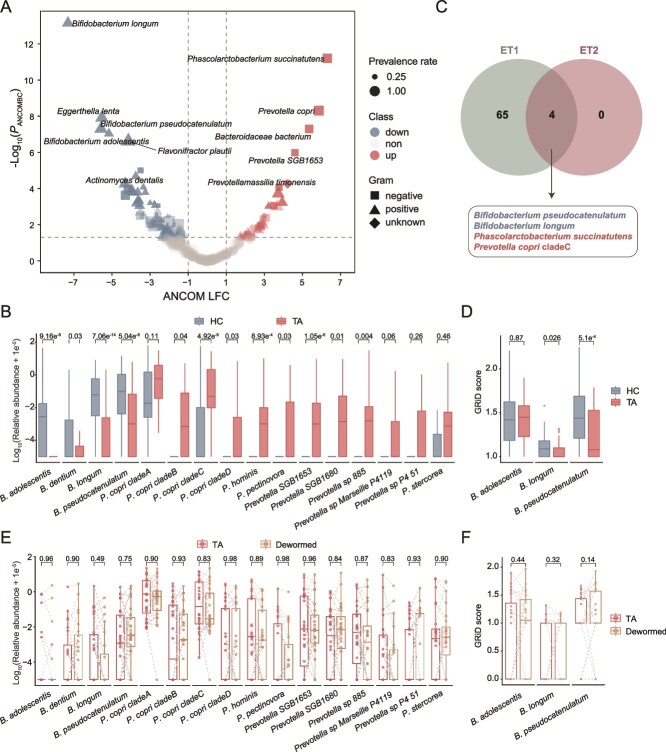
The depletion of *Bifidobacterium* spp. in taeniasis. (A) Differential abundance analysis at the species level. Log_2_-transformed fold changes (LFC) are shown on the X-axis and Log_10_-transformed *P* values are shown on the Y-axis, as determined by the ANCOMBC method. The color indicates the relative abundance of a taxon was unchanged (non), depleted (down), and increased (up) in the patients with *T. asiatica* (TA) compared to those in HCs. (B) Relative abundances of *Bifidobacterium* spp*.* and *Prevotella* spp*.* (C) Venn plot showing the number of bacteria with differential abundances identified in the two enterotypes, respectively. (D) GRiD scores for significantly altered *Bifidobacterium* spp*.* in HC and TA. (E) Paired comparisons of the relative abundances between baseline and post-deworming samples for *Bifidobacterium* spp*.* and *Prevotella* spp*.* identified in the metagenomic analysis. (F) Paired comparisons of GRiD scores between baseline and post-deworming samples from each individual for the three *Bifidobacterium* species. The corrected *P* values for panels B and E were indicated from the results in ANCOMBC analysis. The *P* values for panels D and F were calculated by the unpaired and paired Wilcoxon rank-sum test, respectively. The box plot represents the 25th percentile, median, and 75th percentile and whiskers stretch to 1.5 times the interquartile range from the corresponding hinge.

Our data suggest that tapeworm infection has a significant, enterotype-associated impact on the host gut microbiota, which may persist even after deworming. To further understand how tapeworm infection affects gut microbial changes with respect to enterotypes, we compared differential abundances of taxa between healthy and infected states within each enterotype. We identified 125 microbes with differential abundances between healthy and infected groups: 37 species were increased and 88 species were depleted in the infected group (Fig. 4A). Among these, 60 Gram-positive and 18 Gram-negative species were more abundant in healthy individuals, whereas 14 Gram-positive and 16 Gram-negative species were more prevalent in the infected group (Fig. S8). Five species of *Bifidobacterium*, a major prebiotic in the human gut, showed the most significant decrease in the infected group. Conversely, 8 out of 12 *Prevotella* species exhibited the greatest increase in the infected group (Fig. 4A and B).

We then assessed whether these microbial changes were linked to enterotypes. Using the ANCOMBC2 method [22] to compare taxa abundances in healthy versus infected states for each enterotype, we found only four taxa common to both enterotypes (ET1 and ET2): two depleted *Bifidobacterium* species (*B. pseudocatenulatum* and *B. longum*) and two augmented species (*Phascolarctobacterium succinatutens* and *Prevotella copri* clade C) (Fig. 4C). This suggests that the depletion of *Bifidobacterium* is a consistent feature across enterotypes. To determine if the growth of *Bifidobacterium* was impaired in infected individuals, we measured bacterial replication rates using an *in situ* metagenomic approach (GRiD). The growth rates of *B. pseudocatenulatum* and *B. longum* were significantly reduced in infected individuals compared to HCs (Fig. 4D).

We analyzed the impact of deworming on the gut microbiota, by considering sampling interval time as a fixed effect and individuals as a random effect in ANCOMBC2 analysis. The analysis revealed that the abundance of *Bifidobacterium* spp. and *Prevotella* spp. did not recover post-deworming (Fig. 4E). Similarly, the growth rate of these bacteria, as assessed by GRiD analysis, remained unchanged after deworming (Fig. 4F). This indicates that the marked gut microbiota associated with tapeworm infection may not rapidly revert to its pre-infection state even after parasite clearance. Thus, factors beyond the parasite itself may influence the fitness of these bacteria and exert long-term effects. Consistent with this, only five bacterial species showed differential abundances after deworming (Fig. S9). These findings support the idea that reduced *Bifidobacterium* growth is a hallmark of taeniasis.

### Reduction of *Bifidobacterium* is associated with microbial metabolomic competition

**Figure 5:**
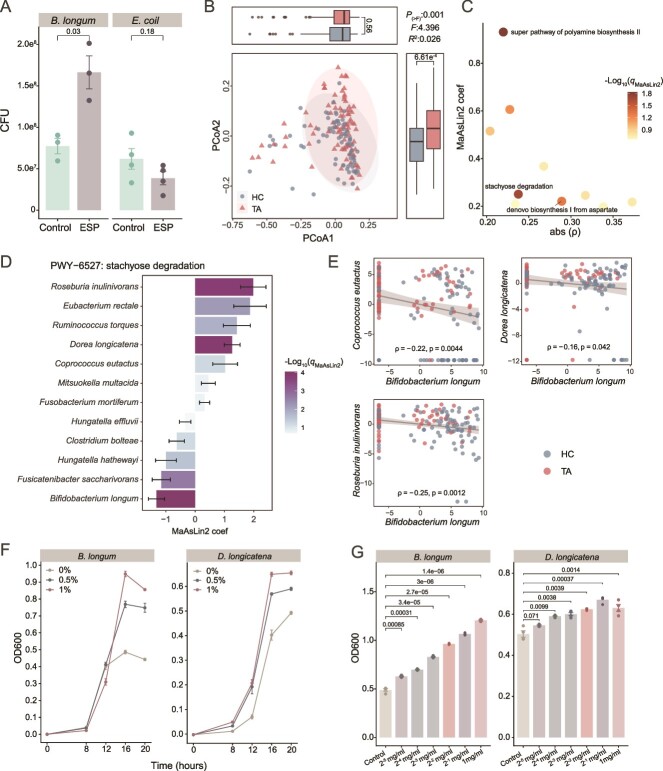
Microbial metabolomic pathway analysis and *in vitro* experiments. (A) The *in vitro* culture of *B. longum* or *E. coli* in the presence of excretory-secretory protein (ESP) (5 mg/ml) collected from different *T. saginata* tapeworm isolates (*n* = 3 or 4). Colony-forming units (CFUs) were counted on plates (*n* = 2 technical replicates for each sample). (B) PCoA using Bray–Curtis distance based on the relative abundances of MetaCyC pathways. (C) The pathways that were significantly increased in infection and negatively correlated with *Bifidobacterium.* The absolute value of the coefficient correlation between each pathway and the abundance of *Bifidobacterium* (*X*-axis) and the coefficient in MaAsLin2 analysis (Y-axis) are shown. Only pathways with *q* value <0.05 in MaAsLin2 analysis were indicated with names. (D) The species with significantly differential abundance in the stachyose degradation pathway (the prevalence >0.1 and *q* value <0.25 after FDR correction) in MaAsLin2 analysis. The error bars indicate the 95% confidence intervals of coefficient estimates. (E) The negative correlations between *B. longum* and other species in the stachyose degradation pathway. The relative abundance was normalized by the centered log-ratio method. (F) *In vitro* growth rate of *B. longum* (left) and *D. longicatena* (right) in the presence of different concentrations (w/v) of stachyose (*n* = 3 replicates for each sample). (G) *In vitro* growth experiments of *B. longum* (left) and *D. longicatena* (right) in the presence of different concentrations (w/v) of ESP (*n* = 3 or 4 replicates). The data for panels A, F, and G are shown for mean ± SD, and *P* values for panels A and G were calculated by Student’s *t*-test. The *R*^2^ statistic and *P* value for panel B were calculated by permutational multivariate analysis of variance (PERMANOVA). The box plot in panel B represents the 25th percentile, median, and 75th percentile and whiskers stretch to 1.5 times the interquartile range from the corresponding hinge.

To gain insights into the factors driving the reduction of *Bifidobacterium*, during tapeworm infection, we investigated whether ES proteins from *T. asiatica* affect the abundance of *Bifidobacterium*. We assessed the impact of these ES proteins on the beneficial bacterium *B. longum*, which had the highest reduction in infection, using *in vitro* experiments. As a control for the beneficial bacterium, we also examined the effect of ES proteins on *E. coli*, a pathobiont that was also depleted during infection. We found that ES proteins from different *T. asiatica* isolates significantly promoted the growth of *B. longum* whereas moderately inhibiting *E. coli* (Fig. 5A). This suggests that the parasite does not directly reduce the fitness of *Bifidobacterium*. Given that *Bifidobacterium* failed to recover even after deworming (Fig. 4E), these findings imply that the reduction of *Bifidobacterium* is more likely due to functional competition from ecological niches within the gut microbiota rather than direct effects of the parasite.

We analyzed the metabolomic functions within the gut microbiome by examining metabolic pathways and gene families. PCoA of gut microbiome metabolic pathways revealed a significant separation between healthy and infected groups, indicating substantial changes in the metabolomic landscape during infection (Fig. 5B). We identified 64 global pathways significantly altered with confounding factors controlled by MaAsLin2 (Table S3). Among these pathways, 10 exhibited a significant negative correlation with the abundance of *Bifidobacterium* taxa. Among them, the stachyose degradation pathway is particularly prominent, as it represents the most implicated pathway that negatively correlated with *Bifidobacterium* (ranked second by FDR) and was increased in infection (Fig. 5C and Table S4). Stachyose is a well-known prebiotic that strongly promotes the growth of *Bifidobacterium* in the human gut. However, the stachyose degradation pathway was markedly upregulated in the infected group and was significantly negatively correlated with 4 of the 5 *Bifidobacterium* species that were depleted during infection (Fig. S10). This unusual link suggests that the increased activity of stachyose degradation by other microbes may contribute to the reduction of *Bifidobacterium* during infection. Specifically, the abundance of the stachyose degradation pathway was significantly higher in seven species, including *Lachnospiraceae* bacteria such as *Coprococcus eutactus*, *Roseburia inulinivorans*, and *Dorea longicatena* (Fig. 5D). We found a negative correlation between the abundance of *B. longum* and some species with increased stachyose degradation pathways (Fig. 5E). *In vitro* experiments confirmed that stachyose promoted the growth of both the stachyose degradation-related bacteria (i.e. *D. longicatena*) and *B. longum* (Fig. 5F). Additionally, ES proteins from the parasite promoted the growth of these bacteria (Fig. 5G). These results raise the possibility that competition for stachyose utilization between *Bifidobacterium* and other microbes potentially contributes to the reduction of *Bifidobacterium* during infection.

**Figure 6:**
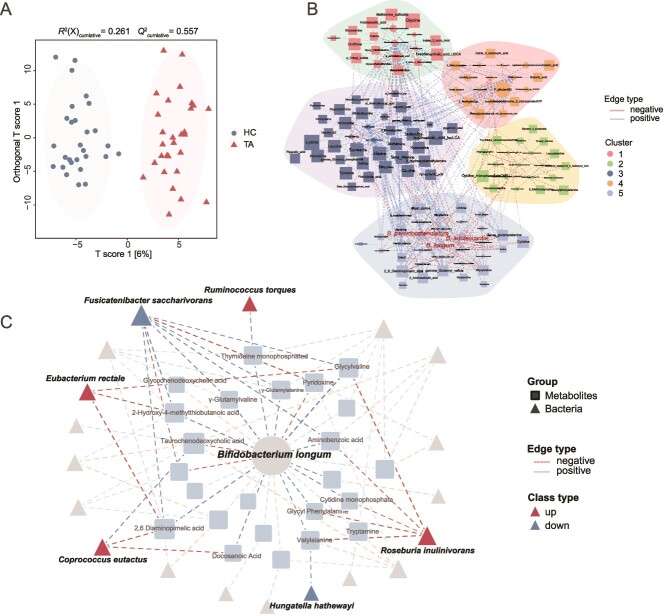
Microbiota-metabolite interaction network in taeniasis. (A) OPLS-DA results for comparing HC and *T. asiatica-*infected (TA) groups. The cumulative *R*^2^ and *Q*^2^ values indicate model fit and predictive ability. (B) The metabolite-microbiota correlation network. Nodes represent bacteria (round) and metabolites (square) and edges indicate positive (red) or negative (blue) correlations. The clusters of closely related bacteria and metabolites relevant to *Bifidobacterium* are highlighted. (C) The metabolite-mediated correlation network focused on *B. longum* and related bacteria involved in the stachyose degradation pathway. Nodes represent *B. longum* (round) and other bacteria (triangle) and metabolites (square) and edges indicate positive (red) or negative (blue) correlations.

We further conducted fecal metabolomic analysis using UHPLC-HRMS on a subset of participants (HC = 28, TA = 29). OPLS-DA analysis of 414 detected metabolites significantly distinguished between healthy and infected groups (Fig. 6A). However, stachyose was almost undetectable in these samples, which might result from the degradation by gut microbes [34, 35]. We identified 110 metabolites with differential concentrations between these groups (Fig. S11). Microbiota-metabolite interaction network analysis revealed that these differential metabolites clustered into five modules closely related to gut microbiota changes during infection (Fig. 6B). *B. longum* was involved in a module with metabolites that showed the most notable changes, including Glycyl-valine, Valyl-alanine, 2-Hydroxy-4-(methylthio) butanoic acid, and 2,6-Diaminopimelic acid (Fig. 6B and C). These metabolites were positively correlated with increased taxa in stachyose degradation pathways, such as *C. eutactus*, *E. rectale*, and *R. inulinivorans* (Fig. 6C). Overall, these findings suggest that the dynamics of gut metabolites are closely linked to the changes in gut microbiota, particularly the reduction of *Bifidobacterium*, during infection.

### Taxa linked with *Bifidobacterium* in stachyose metabolism are implicated in predicting infection

**Figure 7:**
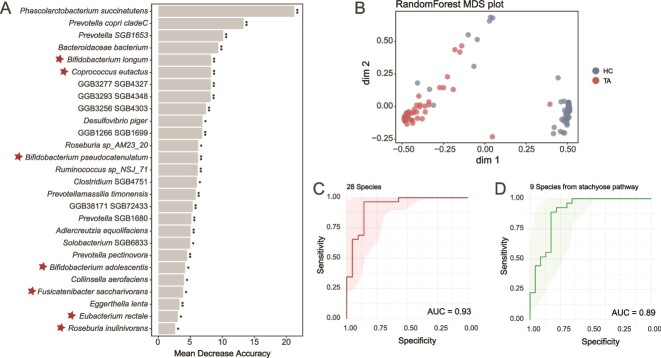
Microbial markers for classifying *T. asiatica* infection from health. (A) The 28 markers were identified in RF analysis for the discovery dataset (*n* = 116). The markers are ranked by mean decrease accuracy and the markers involved in the stachyose degradation pathway were marked with stars. (B) Multidimensional scaling (MDS) plot of proximity matrix based on the 28 optimal markers in randomForest analysis. Receiver operating characteristic (ROC) curves for the validation dataset (*n* = 50) using the 28 optimal markers (C) and the selected 9 optimal markers involved in the stachyose degradation pathway (D). The AUC is shown for each classifier. The statistical significance of each marker in panel A was assessed by permutation testing. **P value < .01; *P value < .05.

Given the significance of the alterations in *Bifidobacterium* and the potential taxa involved in stachyose metabolism, we investigated whether these features could be useful in differentiating individuals with infection. To assess this, we constructed a RF model with a discovery dataset and evaluated its performance with a validation dataset. Through 10-fold cross-validation, we identified 28 taxa with significant differences between the infected and healthy groups (Fig. 7A). Of these, 16 taxa were increased and 12 were decreased following infection, prominently featuring the depleted *Bifidobacterium* species (*n* = 3) and the taxa involved in the increased stachyose degradation pathway (*n* = 5), such as *C. eutactus*, *F. saccharivorans*, and *Roseburia inulinivorans* (Fig. 7A, indicated by stars). The RF classifier effectively distinguished infected individuals from HCs (Fig. 7B), achieving an area under the curve (AUC) of 1.0 in the discovery cohort (*n* = 116) and an AUC of 0.927 in the testing dataset (*n* = 50) (Fig. 7C and Fig. S12A). This suggests that stachyose degradation-related features are strong predictors of taeniasis. In supporting this notion, a new RF model using only the nine bacteria species associated with stachyose degradation also performed well, with an AUC of 1.0 for the discovery dataset and 0.889 for the testing dataset (Fig. 7D and Fig. S12B). However, when applied to predict the health status of individuals after deworming, both models failed to classify these individuals accurately (Fig. S13), indicating a persistent impact of tapeworm infection on the host gut microbiota. These results highlight the significant role of stachyose degradation-related metabolism in predicting and understanding tapeworm infection.

## Discussion

A growing body of literature indicates that parasitic colonization of the host GI tract can significantly alter gut microbiota both structurally and functionally in humans [33, 36, 37]. Despite this, the impact of human taeniasis, a highly prevalent condition globally, remains poorly understood. Our cross-sectional and longitudinal analyses involving 87 patients infected with *T. asiatica* and HCs suggest that *T. asiatica* infection markedly reshapes the gut microbiota across several dimensions. These include microbial diversity, enterotype, metabolic pathway, and metabolite profile, with considerable shifts in the relative abundance of specific bacterial taxa such as species in *Bifidobacterium* and *Prevotella*. Furthermore, our longitudinal data indicate that the gut microbiota in infected patients does not fully revert to the profiles observed in HCs even after successful deworming. This persistence of altered microbiota suggests that *T. asiatica* infection exerts potentially profound and long-lasting effects on the host gut microbiome.

In this study, we found the association between *T. asiatica* infection and the host gut microbiota enterotypes. Enterotypes are distinct clusters of microbial communities in the gut, offering a broad view of gut microbiota composition [27]. Our results reveal that whereas the proportion of infected patients and HCs is balanced within the ET1 enterotype, the ET2 enterotype is significantly enriched among infected patients. Given that enterotypes are associated with carbohydrate and protein metabolism in the gut [38], the predominance of the *Prevotella*-driven enterotype in infected patients suggests a potential change in the metabolomic capabilities of the gut microbiota. As a key genus in the human gut microbiota [39], an increased abundance of *Prevotella* has been linked to various systemic diseases, including insulin resistance [40], and hypertension due to metabolic disorders [41]. Recent studies have shown that individuals with higher *Prevotella* abundance tend to lose more weight when consuming a diet rich in whole grains and fiber, suggesting a role for *Prevotella*-driven enterotypes in obesity management [42]. Moreover, *P. succinatutens*, identified as the most significantly increased bacterial species following *T. asiatica* infection across both enterotypes, is positively correlated with weight loss [43]. Historically, taeniasis was used as a radical weight-loss measure in the 19th century, based on the belief that the parasite directly absorbed nutrients in the GI tract, thereby reducing the host’s weight. Our findings suggest that the gut microbiota alterations induced by *T. asiatica* infection could potentially be associated with weight loss observed in infected individuals, providing new insights into metabolic impact of taeniasis.

Our findings indicate an individual-dependent recovery of gut microbiota following deworming, as compared to HCs. It is hypothesized that clearing the parasites may alter the availability of nutrients or micro-ecological niches in the GI tract of infected patients [44], potentially allowing for changes in gut microbiota compositions. This observation raises an important question: will the gut microbiota of these individuals return to its pre-infection state over time? Our study provides preliminary evidence on this matter, showing that stability after deworming is associated with the baseline gut microbiota diversity and enterotype of each individual. Baseline alpha diversity, which reflects the richness and evenness of microbial species, has been linked to the long-term stability of gut microbial composition in aging and disease contexts [38, 45]. This may be attributed to biodiversity’s role in maintaining ecological balance and stability within microbial ecosystems [46]. Our results suggest that baseline alpha diversity is a predictor of the gut microbiota’s response to deworming. From the perspective of enterotypes, despite the notion that altering human enterotypes is challenging [47], ~41% of individuals who underwent deworming experienced a shift in their enterotypes. This indicates that deworming can have a substantial and individualized impact on gut microbiota composition [48]. Future research should further explore the implications of these changes for patient health and disease management, particularly in the context of personalized medicine.

Another key finding of our study is the significant depletion of *Bifidobacterium* spp. in patients infected with *T. asiatica*, regardless of their enterotype. *Bifidobacterium* spp. are well-recognized for their beneficial roles in the human GI tract [49]. They inhibit the growth of harmful bacteria [50–52], aid in the digestion of dietary fibers [53], and produce short-chain fatty acids (SCFAs) [54] and vitamins [55]. Additionally, *Bifidobacterium* spp. contribute to various health benefits, including alleviating symptoms of irritable bowel syndrome (IBS) [56], inflammatory bowel disease (IBD) [57], and psoriasis [58] through their immunomodulatory effects [59, 60]. Despite the typically asymptomatic nature of taeniasis, the depletion of *Bifidobacterium* spp. highlights a potential health risk [61] and the need to consider exogenous supplementation of these beneficial bacteria in affected patients. Our *in vitro* experiments and metabolomic-based bacteria-metabolic network analysis suggest that the reduction in *Bifidobacterium* spp. during infection may be driven by microbial interactions mediated through metabolites, rather than by *T. asiatica* directly. The interactions and competition between gut microbiota are known to shape the gut ecosystem both structurally and functionally [62]. Our findings suggest that competition for resources, particularly stachyose, might impair *Bifidobacterium* growth. Specifically, other microbes such as *C. eutactus*, *R. inulinivorans*, and *D. longicatena*, which are adept at utilizing stachyose, might be contributing to this competitive exclusion. In addition, our results also suggest Gram-positive bacteria are more likely to be affected by infection, implying an underlying mechanism related to the differences in bacterial cell walls and cell membranes. Thus, we cannot exclude the possibility that the underlying mechanisms might vary among microbe species and the reductions for some Gram-positive microbes are possible via a membrane-damaging action during the tripartite interactions between the parasite, the gut microbiota, and the host. Future research, incorporating both *in vivo* and *in vitro* experiments, is necessary to further elucidate the complex interactions in taeniasis.

## Conclusions

This study describes the intestinal micro-ecology landscape of *T. asiatica-*infected patients using metagenomics and metabolomics. Our findings demonstrate significant alterations in gut micro-ecology, even in asymptomatic infections, including shifts in enterotypes and reductions in beneficial prebiotics. These changes may pose long-term health risks and can persist for several months after deworming, varying among individuals. Thus, we recommend that healthcare providers carefully evaluate and monitor the intestinal micro-ecology of patients both during infection and after deworming.

## Supplementary Material

Supplementary_Figures_1-13_wrae213

Supplementary_tables_1-4_wrae213

Supplementary_information_wrae213

## Data Availability

Raw metagenomic data for all samples used in this study have been deposited in the Sequence Read Archive (SRA) under project accession no. PRJNA1148078. Original R scripts, metadata, and OTU tables are available on GitHub (https://github.com/Axolotl233/Tapeworm-gut_microbiome).
