# Supplementary material for: Taeniasis impacts human gut microbiome composition and function
Source: ISME J. 2024 Oct 23;18(1):wrae213. doi: 10.1093/ismejo/wrae213 (PMC11536184; doi:10.1093/ismejo/wrae213)
Supplement: Supplementary_Figures_1-13_wrae213 [file supplementary_figures_1-13_wrae213.pdf]

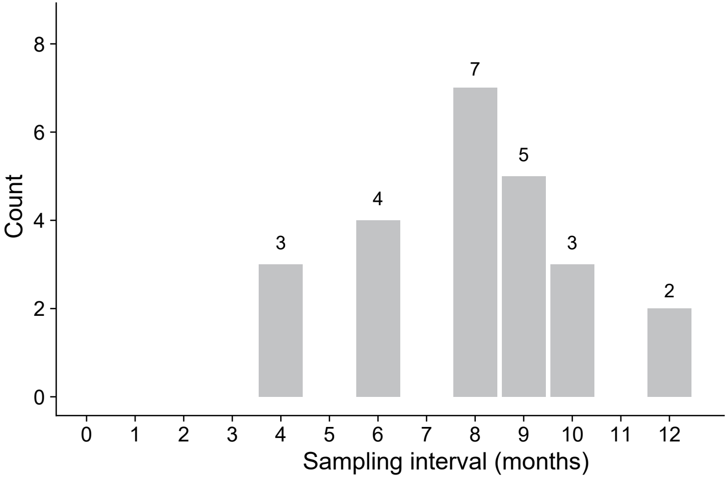

Fig. S1. Distribution of time interval between baseline and dewormed samples from the same individual.

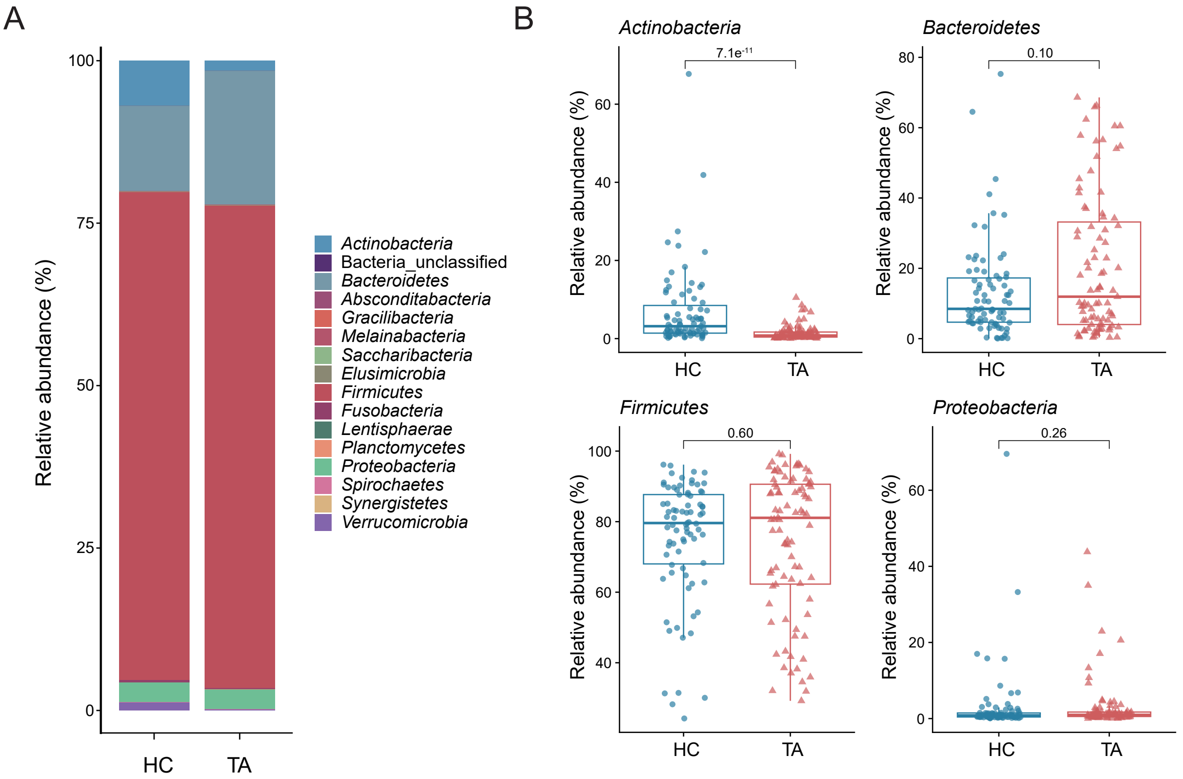

Fig. S2. Relative abundances of the gut microbiome at the phylum level in infection of *T. asiatica*. (A) The mean relative abundance of each phylum in the HC and TA groups. **(B)** Comparison of relative abundance of top 4 phyla between HC and TA. The *P* values were calculated by the Wilcoxon rank sum test. The box plot represents the 25th percentile, median, and 75th percentile and whiskers stretch to 1.5 times the interquartile range from the corresponding hinge.

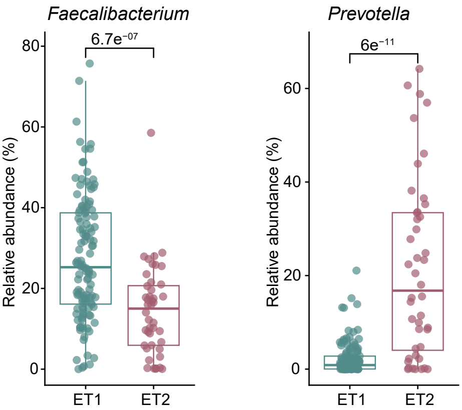

Fig. S3. Relative abundances of *Faecalibacterium* and *Prevotella* within the enterotypes. The *P* values were calculated by Wilcoxon rank sum test. The box plot represents the 25th percentile, median, and 75th percentile and whiskers stretch to 1.5 times the interquartile range from the corresponding hinge.

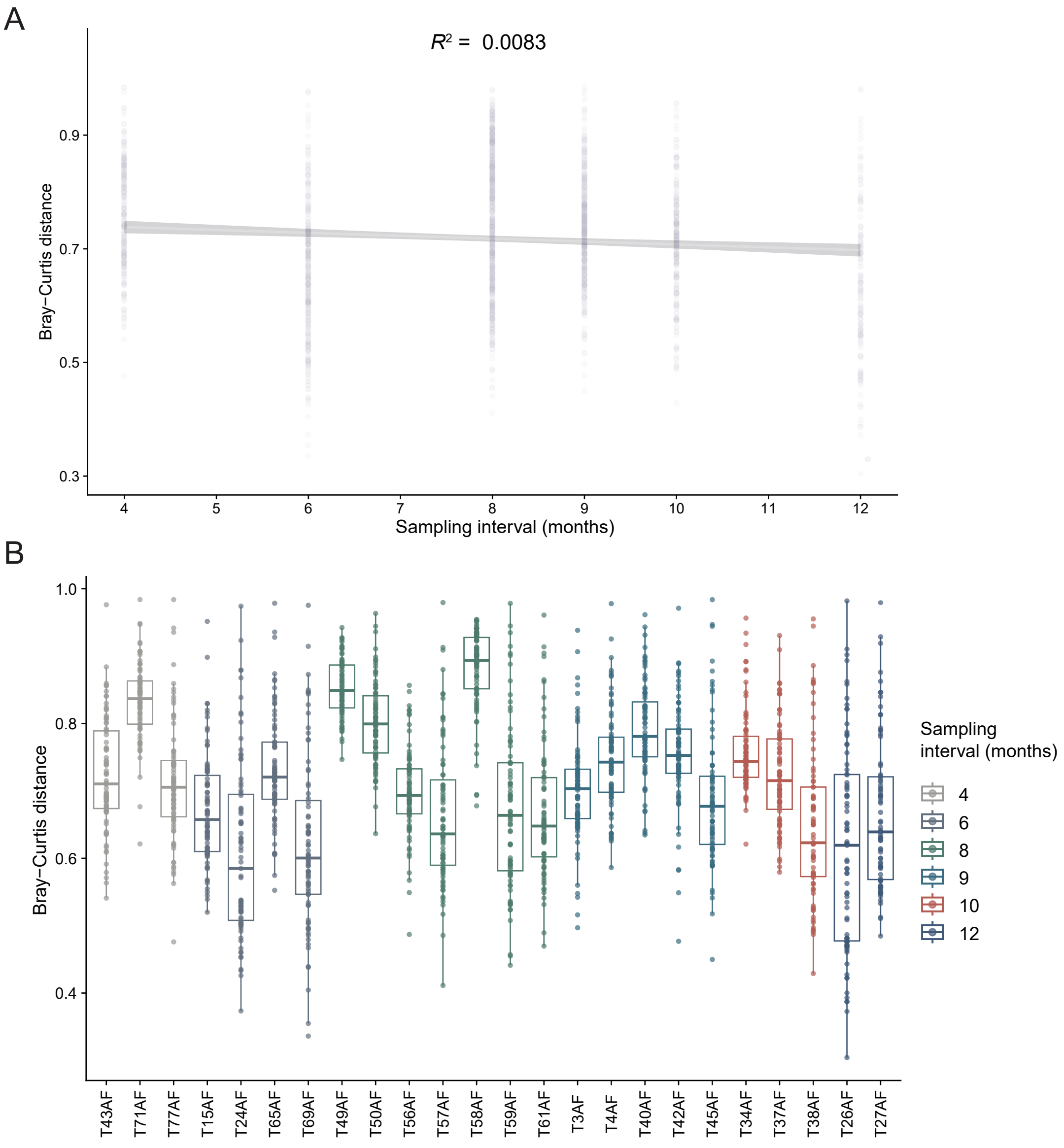

Fig. S4. Bray-Curtis distances of the gut microbiome between HC and post-deworming individuals. (A) Linear regression of Bray-Curtis distances for dewormed samples to healthy control (HC) on sampling intervals. **(B)** The distribution of Bray-Curtis distances to each HC for each dewormed individual. The box plot represents the 25th percentile, median, and 75th percentile and whiskers stretch to 1.5 times the interquartile range from the corresponding hinge. The correlation was calculated by the Spearman rank correlation coefficient.

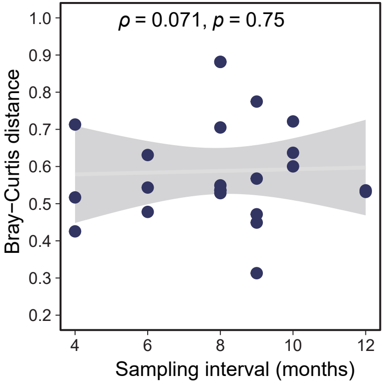

Fig. S5. Correlation analysis between paired Bray-Curtis distances and sampling intervals within individuals. The correlation was calculated by spearman rank correlation coefficient.

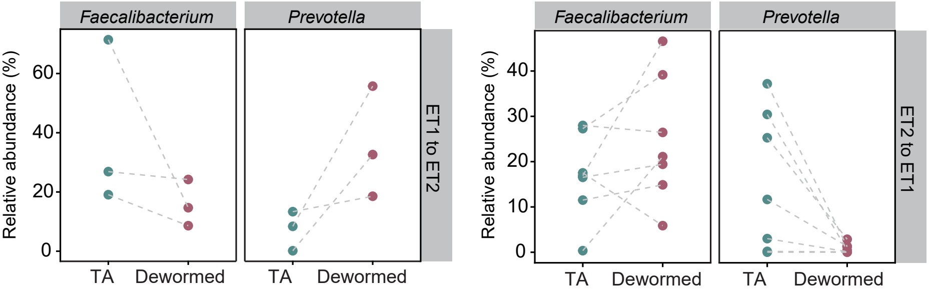

Fig. S6. Paired comparisons of relative abundances of *Faecalibacterium* and *Prevotella* between baseline and dewormed samples. The enterotype switch is indicated.

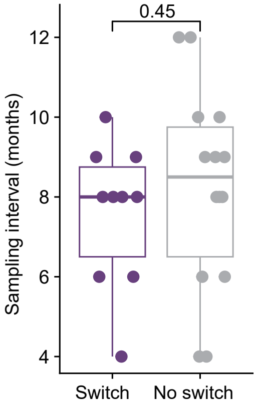

Fig. S7. Comparisons of sampling intervals between samples with (n = 10) and without (n = 14) enterotype shifts following deworming. The *P* value was determined by paired Wilcoxon rank sum test. The box plot represents the 25th percentile, median, and 75th percentile and whiskers stretch to 1.5 times the interquartile range from the corresponding hinge.

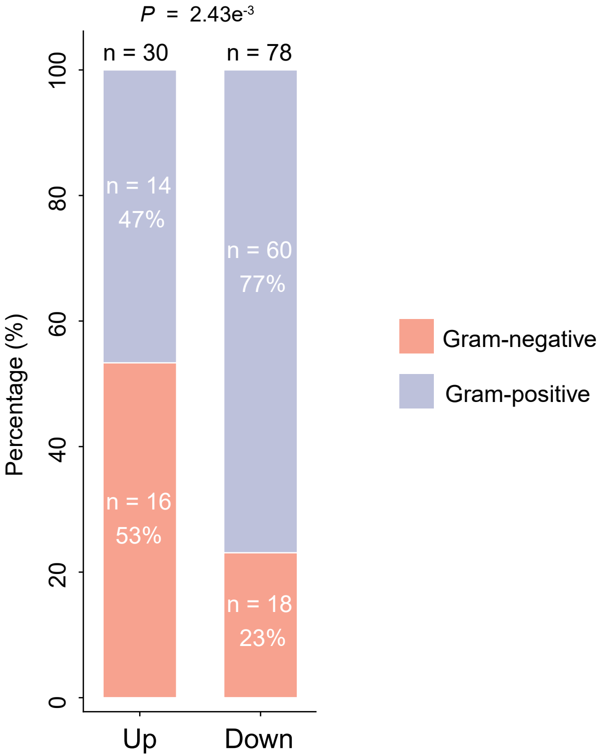

Fig. S8. Distribution of Gram-positive and Gram-negative bacteria among the differentially abundant taxa in infection. The *P* value was calculated by chi-square test.

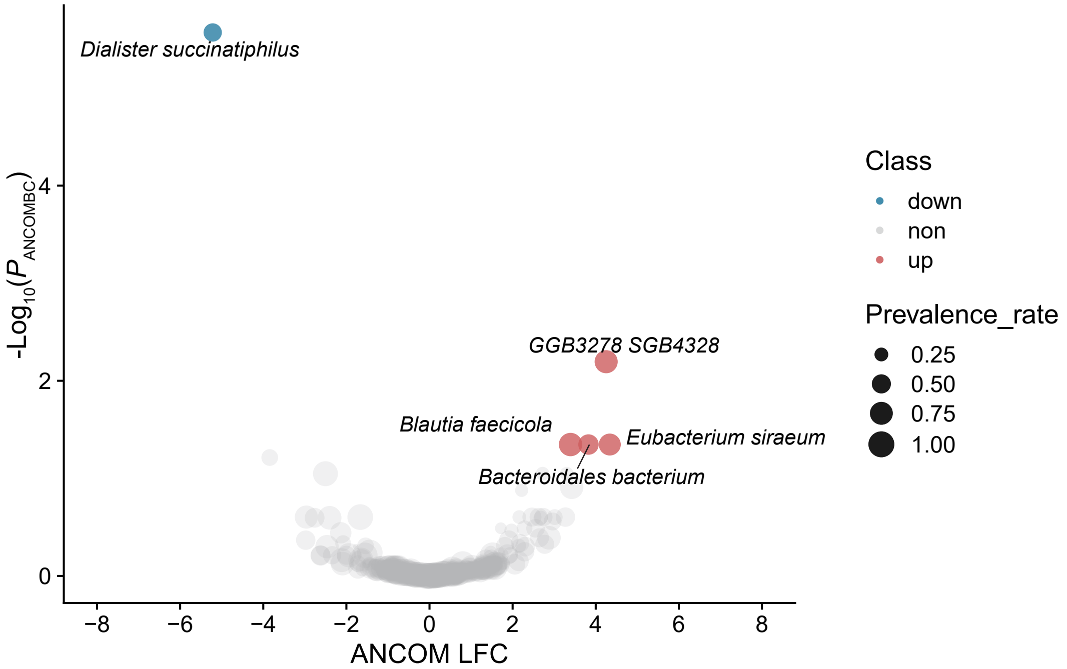

Fig. S9. Differential abundance analysis at the species level. Log2-transformed fold changes (LFC) are shown on the X-axis and Log10-transformed *P* values are shown on the Y-axis, as determined by the ANCOMBC method. The color indicates the relative abundance of a taxon was unchanged (non), depleted (down), and increased (up) in the post-deworming samples compared to baseline samples.

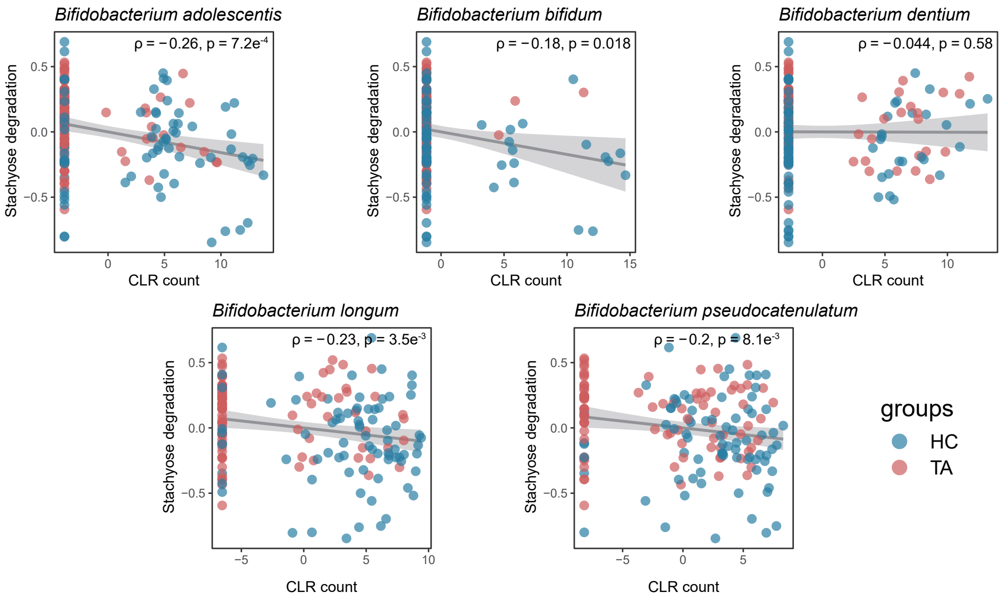

Fig. S10. Spearman correlations between *Bifidobacterium* spp. and stachyose degradation pathway. The relative abundance of bacteria was normalized by the centered log-ratio method.

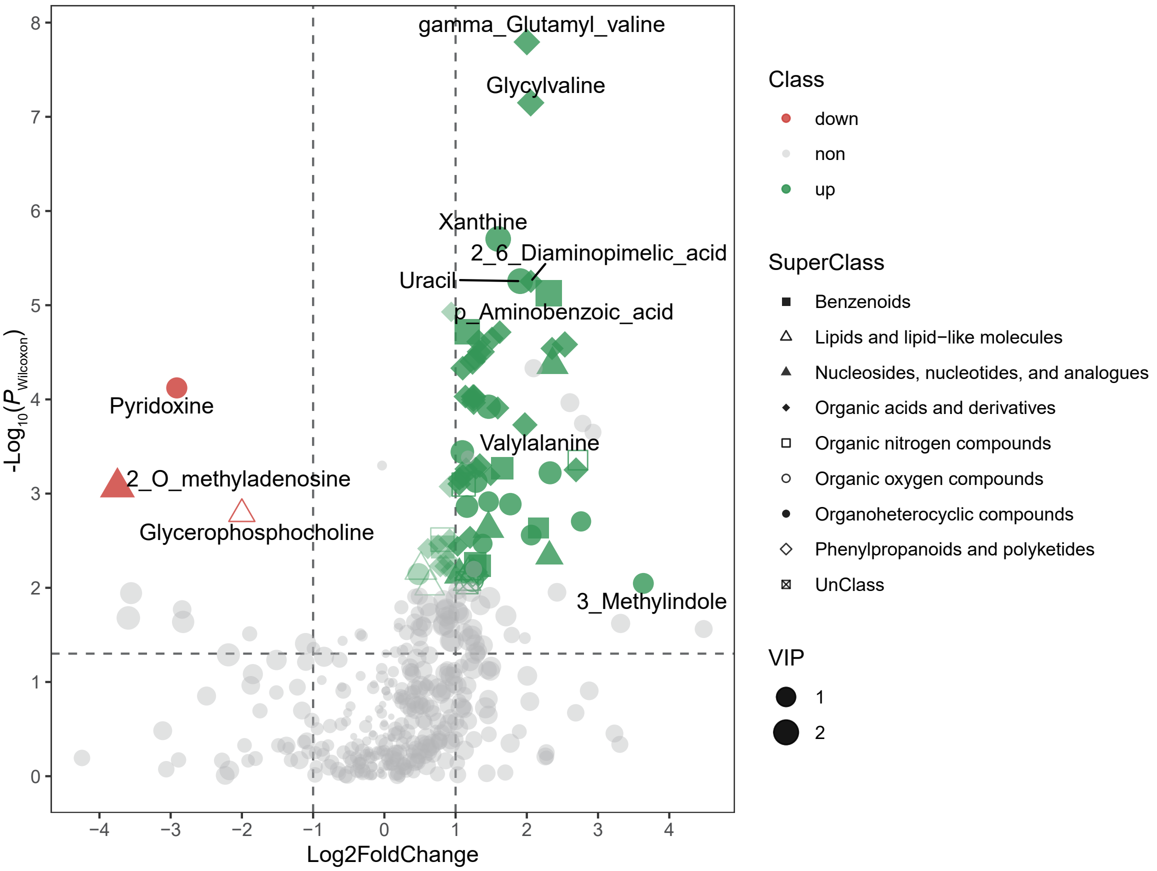

Fig. S11. Differential analysis for metabolite. Log2-transformed fold changes (LFC) are shown on the X-axis and Log10-transformed *P* values are shown on the Y-axis, as determined by the wilcoxon rank sum test and adjusted by Benjamini-Hochberg method. The color indicates the concentration of a metabolite was unchanged (non), depleted (down), and increased (up) in the *T. asiatica* infected patients.

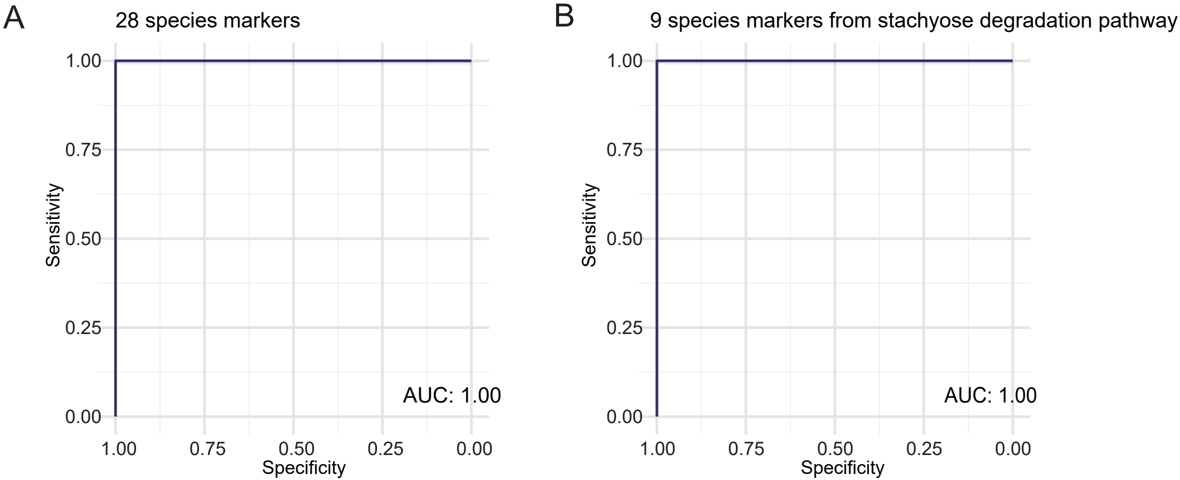

Fig. S12. Area under the curve (AUC) for each Random Forest classifier in predicting *T. asiatica* infection for the training dataset. **(A)** The test based on the 28 optimal markers. **(B)** The test based on the 9 optimal markers involved in the stachyose degradation pathway.

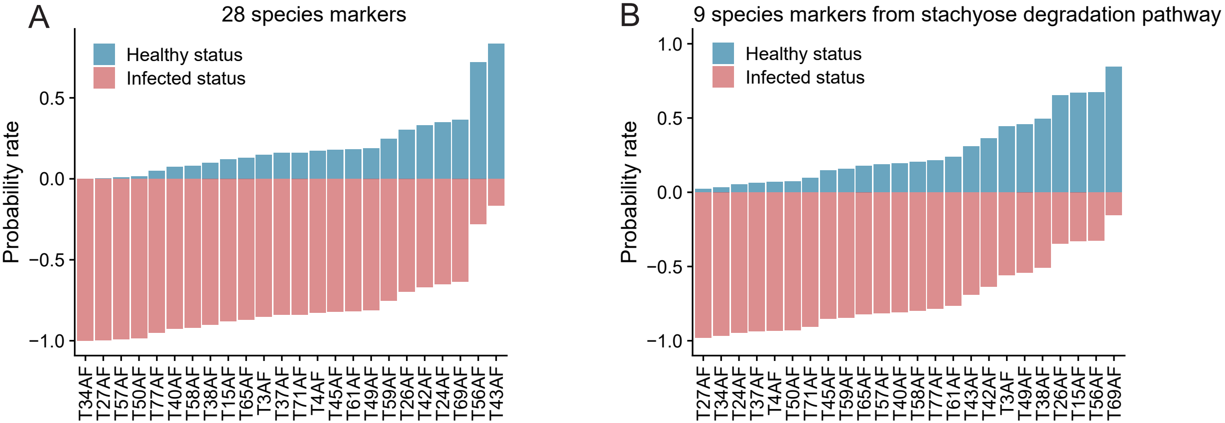

Fig. S13. Probability of health status predicted by Random Forest classifiers for dewormed individuals. (A) The model based on the 28 optimal markers. **(B)** The model based on the 9 optimal markers involved in the stachyose degradation pathway. The Y-axis indicates the probability for healthy status (blue) or infected status (red).
