# Supplementary material for: Taeniasis impacts human gut microbiome composition and function
Source: ISME J. 2024 Oct 23;18(1):wrae213. doi: 10.1093/ismejo/wrae213 (PMC11536184; doi:10.1093/ismejo/wrae213)
Supplement: Supplementary_information_wrae213 [file supplementary_information_wrae213.docx]

**Supplementary information**

**Supplementary methods**

**Deworming procedures**

A traditional remedy [1] with a combination of pumpkin and betel nut was performed as follows: patients first consumed 100 g of pumpkin seeds on an empty stomach, accompanied by at least 800 mL of water. One hour later, they took 300 mL of a decoction made from 120 g of betel nut. After another hour, they ingested 250 mL of 20% mannitol for catharsis and monitored whether the tapeworm was excreted in the feces.

**Metabolomic analysis**

The preparations of the samples for UHPLC-HRMS-based metabolomic analysis were conducted using the method previously reported [2]. Briefly, the fecal samples of each participant were homogenized in liquid nitrogen and vortexed with cold extraction solvent. The stock solutions of stable-isotope internal standards were added to absolutely quantify each metabolite in targeted metabolomics. Then, the prepared samples were incubated on ice for 20 min and centrifuged for 20 min (14,000 × g, 4°C). The supernatants were collected and further processed through 96-well protein precipitation plates. The obtained eluent was then dried using a vacuum centrifuge at 4°C. For liquid chromatography-mass spectrometry (LC-MS) analysis, the dried samples were reconstituted in 100 μL of an acetonitrile/water solution, transferred to LC vials and were then analyzed using a quadrupole time-of-flight mass spectrometer (QTRAP-MS, 6500+, Sciex) interfaced to hydrophilic interaction chromatography via electrospray ionization. LC separation was conducted on an ACQUITY UPLC BEH Amide column using a gradient of solvent A (25 mM ammonium acetate and 25 mM ammonium hydroxide in water) and solvent B (acetonitrile). The QTRAP-MS was operated in both positive and negative ionization modes. During MS acquisition, the instrument was configured to scan within an m/z range 60–1,000 Da, with the accumulation time for the time-of-flight (TOF) MS scan set at 0.20 s per spectrum. The product ion scan was acquired using information-dependent acquisition (IDA) with a high-sensitivity mode selected.

1. Li T, Ito A, Chen X, Long C et al. Usefulness of pumpkin seeds combined with areca nut extract in community-based treatment of human taeniasis in northwest Sichuan Province, China. *Acta Trop* 2012; **124**: 152–57.

2. Lv D, Cao X, Zhong L et al. Targeting phenylpyruvate restrains excessive NLRP3 inflammasome activation and pathological inflammation in diabetic wound healing. *Cell Rep Med* 2023; **4**: 101129.

**Supplementary Files**

**Fig. S1. Distribution of time interval between baseline and dewormed samples from the same individual.**

**Fig. S2. Relative abundances of the gut microbiome at the phylum level in infection of *T. asiatica*. (A)** The mean relative abundance of each phylum in the HC and TA groups. **(B)** Comparison of relative abundances of top 4 phyla between HC and TA. The *P* values were calculated by Wilcoxon rank sum test. The box plot represents the 25th percentile, median, and 75th percentile and whiskers stretch to 1.5 times the interquartile range from the corresponding hinge.

**Fig. S3. Relative abundances of *Faecalibacterium* and *Prevotella* within the enterotypes.** The *P* values were calculated by Wilcoxon rank sum test. The box plot represents the 25th percentile, median, and 75th percentile and whiskers stretch to 1.5 times the interquartile range from the corresponding hinge.

**Fig. S4. Bray-Curtis distances of the gut microbiome between HC and post-deworming individuals.** (**A**) Linear regression of Bray-Curtis distances for dewormed samples to healthy control (HC) on sampling intervals. (**B**) The distribution of Bray-Curtis distances to each HC for each dewormed individual. The box plot represents the 25th percentile, median, and 75th percentile and whiskers stretch to 1.5 times the interquartile range from the corresponding hinge. The correlation was calculated by the Spearman rank correlation coefficient.

**Fig. S5. Correlation analysis between paired Bray-Curtis distances and sampling intervals within individuals.** The correlation was calculated by Spearman rank correlation coefficient.

**Fig. S6. Paired comparisons of relative abundances of *Faecalibacterium* and *Prevotella* between baseline and dewormed samples.** The enterotype switch is indicated.

**Fig. S7. Comparisons of sampling intervals between samples with (n = 10) and without (n = 14) enterotype shifts following deworming.** The *P* value was determined by paired Wilcoxon rank sum test. The box plot represents the 25th percentile, median, and 75th percentile and whiskers stretch to 1.5 times the interquartile range from the corresponding hinge.

**Fig. S8. Distribution of Gram-positive and Gram-negative bacteria among the differentially abundant taxa in infection.** The *P* value was calculated by chi-square test.

**Fig. S9. Differential abundance analysis at the species level.** Log2-transformed fold changes (LFC) are shown on the X-axis and Log10-transformed *P* values are shown on the Y-axis, as determined by the ANCOMBC method. The color indicates the relative abundance of a taxon that is unchanged (non), depleted (down), and increased (up) in the post-deworming samples compared to baseline samples.

**Fig. S10. Spearman** **correlations between *Bifidobacterium* spp. and stachyose degradation pathway.** The relative abundance of bacteria was normalized by the centered log-ratio method.

**Fig. S11. Differential analysis for metabolites.** Log2-transformed fold changes (LFC) are shown on the X-axis and Log10-transformed *P* values are shown on the Y-axis, as determined by the Wilcoxon rank sum test and adjusted by Benjamini-Hochberg method. The color indicates the concentration of a metabolite was unchanged (non), depleted (down), and increased (up) in the *T. asiatica-*infected patients.

**Fig. S12. Area under the curve (AUC) for each Random Forest classifier in predicting *T. asiatica* infection for the training dataset.** (**A**) The test based on the 28 optimal markers. (**B**) The test based on the 9 optimal markers involved in the stachyose degradation pathway.

**Fig. S13. Probability of health status predicted by Random Forest classifiers for dewormed individuals.** (**A**) The model based on the 28 optimal markers. (**B**) The model based on the 9 optimal markers involved in the stachyose degradation pathway. The Y-axis indicates the probability for healthy status (blue) or infected status (red).

**Table S1. Metadata of the cohort in this study.**

**Table S2. Spearman correlation coefficient between the abundance of each microbe at baseline and paired Bray-Curtis distance within individuals.**

**Table S3. Pathways with significantly differential abundance between HC and TA in MaAsLin2 analysis.** Only results for the taxa with a prevalence > 0.1 and q value < 0.25 after FDR correction are shown.

**Table S4. Spearman correlation between each pathway and *Bifidobacterium*.** *P* values were adjusted using the 'BH' method and only pathways with p.adj < 0.05 are shown.
